# Erianin inhibits the progression of triple-negative breast cancer by suppressing SRC-mediated cholesterol metabolism

**DOI:** 10.1186/s12935-024-03332-2

**Published:** 2024-05-11

**Authors:** Ming Li, Shiyao Kang, Xuming Deng, Huimin Li, Yuan Zhao, Wenru Tang, Miaomiao Sheng

**Affiliations:** 1 Laboratory of Molecular Genetics of Aging & Tumor, Medical School, Kunming University of Science and Technology, Kunming, Yunnan, 650500 China; 2 Kunming University of Science and Technology Affiliated Puer City People’s Hospital, Puer, Yunnan, 665000 China

**Keywords:** Erianin, TNBC, SRC, Cholesterol levels, Molecular mechanism

## Abstract

Triple-negative breast cancer (TNBC) is highly malignant and lacks effective biotherapeutic targets. The development of efficient anticancer drugs with low toxicity and few side effects is a hotspot in TNBC treatment research. Although erianin is known to have potent antitumor activity, its regulatory mechanism and target in TNBC have not been fully elucidated, hampering further drug development. This study showed that erianin can significantly inhibit TNBC cell proliferation and migration, promote cell apoptosis, and inhibit the growth of transplanted tumors in mice. Mechanistically, through network pharmacology analysis, molecular docking and cellular thermal shift assays, we preliminarily identified SRC as the cellular target of erianin. Erianin potently inhibited the expression of SRC, which mediated the anticancer effect of erianin in TNBC. Moreover, erianin can downregulate the expression of genes related to cholesterol synthesis and uptake by targeting SRC, interfering with cholesterol levels in TNBC, thereby inhibiting the progression of TNBC in vivo and in vitro. Taken together, our results suggest that erianin may inhibit the progression of TNBC by suppressing SRC-mediated cholesterol metabolism, and erianin has the great potential to be an effective treatment for TNBC patients.

## Introduction

Breast cancer (BC) is the most commonly diagnosed cancer among women and poses a significant threat to their health. According to the latest worldwide cancer statistics of 2020, BC accounted for the highest number of new cases (2.26 million) among cancers, surpassing lung cancer (2.2 million) [1]. In China, BC contributes 12.2% and 9.6% of the world’s total annual number of new cancer cases and related deaths, respectively [2]. TNBC is a subtype of BC that accounts for 10–20% of invasive BC cases [3]. Because TNBC patients do not express the estrogen receptor, progesterone receptor or human epidermal growth factor receptor 2 (Her-2) and lack clear therapeutic targets, they often cannot benefit from endocrine therapy or anti-Her-2-targeted therapy. In addition, TNBC is characterized by high metastasis and recurrence rates [4–6]. Currently, the combination of taxane and anthracycline is a popular option for treating TNBC clinically. However, anthracycline causes irreversible toxic damage to the heart, which is difficult for many patients to tolerate. Moreover, once patients develop chemotherapy drug resistance, tumor relapse occurs, and the tumor metastasizes rapidly [7, 8]. Therefore, the research and development of anticancer drugs with high efficacy and low toxicity has become an interesting topic and a difficult goal in the treatment of TNBC.

Reprogramming of lipid metabolism is a hallmark of cancer [9, 10]. Cholesterol, an important component of blood lipids, is thought to be fundamental to cancer cell proliferation and survival [11]. In addition to being a constituent of the cell membrane, cholesterol is also a precursor to bile acids, steroids and vitamin D and plays a crucial role in cell growth and differentiation. Moreover, cholesterol is distributed to a large extent in lipid rafts, which are small regions in the cell membrane that act as cellular signal transducers [12]. Cholesterol homeostasis in mammalian cells is maintained by the regulation of de novo synthesis, uptake, efflux, and storage processes [13]. Multiple cancers have been found to have enhanced cholesterol biosynthesis, which promotes tumor growth, metastasis, stem cell maintenance, and resistance to treatments [11, 14]. Cholesterol and its metabolites were discovered to preclinically and clinically enhance tumor progression in patients with BC. In comparison to other subtypes, TNBC demonstrates increased cholesterol biosynthesis, which may have profound biological effects and provide ideas for potential therapeutic approaches [13].

Increasing amounts of attention are being given to researching the study and application of substrates derived from natural plants. Erianin (2-methoxy-5-[2-(3,4,5-trimethoxy phenyl) ethyl]-phenol), a bibenzyl compound, is an active compound extracted from Dendrobium. It has numerous biological functions, including programmed cell death induction, angiogenesis inhibition, and antioxidant and antitumor effects. Previous studies have shown that erianin can inhibit the growth of tumor cells by inducing apoptosis [15, 16], autophagy [17, 18], ferroptosis [19] and other pathways. Sheng et al. [20] found that erianin could exert its anti-liver cancer effect by inhibiting the activity of pyruvate carboxylase. Chen P et al. [19] found that erianin could inhibit the proliferation and migration of lung cancer cells via calcium/calmodulin-dependent ferroptosis. At present, little is known about the antitumor effects of erianin on human BC cells in the BC setting, and it has been reported that erianin can inhibit the proliferation and migration of T47D cells and can also induce cell apoptosis [21]. Erianin and its derivative (Ecust004) can suppress BC cell growth, invasion, and migration via EMT regulation [22]. Erianin induces apoptosis in TNBC cells by inhibiting the PI3K/Akt pathway [23]. However, the molecular mechanism of action and the drug targets of erianin in TNBC are unclear, which limits the further development of this natural anticancer product.

Thus, in the present study, we explored the effects of erianin on TNBC and its underlying mechanisms. Erianin has been shown to significantly inhibit the proliferation and migration of TNBC cells and the growth of transplanted tumors. Mechanistically, our study revealed that erianin may inhibit the progression of TNBC by downregulating the expression of SRC and interfering with cholesterol metabolism. Overall, we showed that erianin exhibited potent anticancer efficacy both in vitro and in vivo and has great potential to be developed as an effective therapeutic agent for TNBC patients.

## Materials and methods

### Materials

Purified erianin (HPLC, ≥ 98%) was purchased from Shanghai Yuanye Biotechnology Co., Ltd. (Shanghai, China, B20844). A stock solution of 800 µM was made in dimethyl sulfoxide (DMSO, D1435, Sigma, USA) and stored in the dark at -80 °C. A CCK-8 detection kit was purchased from RiboBio (C-005), goat serum was purchased from Jackson (005-000-121), and an immunohistochemical secondary antibody and DAB developer were purchased from Dako (K5007). SRC (2109 S), as well as secondary antibodies, were purchased from Cell Signaling Technology (Beverly, California, USA). GAPDH (AC001) was purchased from ABclonal (Massachusetts, USA). Vinculin (bsm-54148R) was purchased from Bioss(BeiJing, China), PP2(HY-13,805) was purchased from MCE (New Jersey, USA), 7.5% and 10% PAGE Gel Fast Preparation Kit (PG111 and PG112) were purchased from Shanghai Epizyme Biomedical Technology Co., Ltd. (Shang Hai, China), alanine aminotransferase assay kit (C009-3-1), aspartate aminotransferase assay kit (C010-1-1) and Total cholesterol assay kit (A111-1-1) were purchased from Nanjing Jiancheng Bioengineering Institute (Nan Jing, China), High-sig ECL Western Blotting Substrate (80-5001) was purchased from Tanon (Shang Hai, China), modified sodium citrate antigen repair solution (P0083), Cholesterol (≥ 99%, ST1155), RIPA Lysis Buffer (P0013B) and PMSF (ST505) were purchased from Beyotime Biotechnology (Shanghai, China), EndoFree Maxi Plasmid Kit (DP117) and BCA Protein Assay Kit (X0927) were purchased from TIANGEN (Bei Jing, China), bovine serum albumin (BSA) was purchased from Sigma‒Aldrich(9048-46-8), Annexin-V-FLUOS Statining Kit was purchased from Roche (Germany), TransIntro EL transfection reagent (FT201) was purchased from TransGen Biotech(Beijing, China). FBS (10,099,141), RPMI-1640 (C11875500BT), Opti-MEM™ (31,985,070) and DMEM (C11995500BT) were purchased from Thermo Fisher Scientific.

### Cell culture

The MDA-MB-231 and 4T1 cell lines were acquired from the Cell Culture Center of Peking Union Medical College, Beijing. The cell lines were incubated in humidified incubators at 37 °C and 5% CO2. 4T1 cells were cultured in RPMI-1640 medium supplemented with 10% FBS. MDA-MB-231 cells (triple-negative/basal-like BC cells) were cultured in DMEM supplemented with 10% FBS.

### Detection of cell proliferation

A CCK-8 assay was used to evaluate the effect of drug treatment on the proliferation and viability of MDA-MB-231 and 4T1 cells. The cells were inoculated into a 48-well plate at a concentration of 2 × 10^4^ cells per well. When the cells were 70–80% confluent, they were treated with different drugs. After the treatment was completed, 20 µl of CCK-8 solution was added to each well at different times, and an enzyme labeling instrument was used to determine the absorbance at 450 nm. Cell survival rate (%) = (D experimental group-D blank group)/(D negative control group-D blank group)×100%.

### Apoptosis detection

A propidium iodide (PI)/annexin V-FITC kit was used to detect apoptosis in two TNBC cell lines. MDA-MB-231 and 4T1 cells were plated in 6-well cell culture plates at 1 × 10^5^ cells/well and incubated to achieve adherence. The cells were treated with DMSO (control) or erianin for 24 h, digested with 0.25% trypsin without EDTA, washed three times with 1× phosphate buffer saline (PBS) and finally resuspended in 1× combined buffer. Then, Annexin V-FITC/PI double staining and an Annexin V-FITC/PI apoptosis detection kit was used for staining at room temperature for 10 min. Then, the cells were analyzed by a C6 flow cytometer (USA, BD). Approximately 100,000 counts were made for each sample, and the percentage of apoptotic cells was calculated.

### Cell transfection

The pcDNA3.1-SRC vector (General Biosystems, Anhui, China) was transfected into MDA-MB-231 cells using the TransIntro EL transfection reagent to determine the role of SRC. When the cell confluence reached 70%, the cell culture medium was removed, and Opti-MEM was used to mix the plasmid with TransIntro EL transfection reagent (with pcDNA3.1 empty plasmid in the control group and with pcDNA3.1-SRC plasmid in the experimental group); the mixture was incubated for 15–20 min at room temperature without light and then added to the cell petri dish. The cells were subsequently transferred to the cell incubator. The medium was replenished with complete medium after 4–6 h, and the cells were collected for follow-up experiments after 24 h.

### Transwell assays

After TNBC cells were treated with 40 nM erianin for 24 h, the cells were digested and counted, and the cells were mixed with medium containing 1% FBS. Subsequently, a 24-well plate was prepared, and complete medium (500 µL) was added to the lower chamber. Additionally, 1% FBS medium (200 µL) was added to each well of the upper chamber, containing approximately 1 × 10^4^ cells per well. The 24-well plate was removed after 16 h, and the liquid in the upper chamber was removed, followed by the addition of PBS and two rounds of washing. The nonmigratory cells were removed using a cotton swab. The remaining cells were fixed in 4% paraformaldehyde and stained with 1% hematoxylin for cells that had migrated to the lower surface. Observations of cell migration were made and recorded under a microscope after staining.

### Animal experiment

BALB/c mice and BALB/c nude mice were purchased from Yunnan University Experimental Animal Center, Kunming University of Science and Technology. We chose 6- to 8-week-old female mice for the experiment. The mice were housed under a specific pathogen-free (SPF) grade experimental system. 4T1 cells were injected into each BALB/c mouse at 3 × 10^5^, and MDA-MB-231 cells were injected into each BALB/c nude mouse at 3 × 10^5^ on the second left mammary fat pad. When the tumor size reached approximately 15 mm^3^, the mice were randomly divided into a control group and an experimental group, and the body weights of the mice were recorded. The mice in the experimental group were injected intraperitoneally with erianin at 4 mg/kg or PP2 at 8 mg/kg. The control group mice received an injection of the same volume of solvent (1% DMSO). When the mice were treated with cholesterol, 8 mg/kg cholesterol was given to the mice, and the control group was given the same volume of solvent (1% methanol). The treatments mentioned above were administered every two days, whereas the longest and shortest tumor diameters were measured using Vernier calipers, and the analysis was performed every two days. The tumor volume was calculated as follows: tumor volume = long diameter × short diameter^2^/2. After the completion of the experiment (BALB/c nude mice were treated for 21 days and BALB/c mice for 17 days), the mice were deeply anesthetized with 3% pentobarbital sodium and weighed. The mice were killed by cervical dislocation after blood was drawn from their eyeballs, after which the tumors were dissected, photographed, and weighed. All protocols were approved by the Animal Ethics Committee of Kunming University of Science and Technology (PZWH K2019-0005).

### Hematoxylin and eosin (HE) assays

The liver tissue taken from the mice was placed in a 10% neutral formalin solution for 24 h, fixed for 24 h, and washed with running water for 24 h. Then, the tissue was subjected to alcohol gradient dehydration, xylene transparency, wax soaking, and embedding. The embedded wax blocks were fixed on a slicer to a thickness of 5 μm. The slices were dewaxed and rehydrated for 5 min after hematoxylin staining. After the excess hematoxylin was washed with tap water, hydrochloric acid and alcohol differentiation were carried out for 1 s, after which the ammonia was returned to blue. Three minutes later, the sections were stained with eosin, rinsed with tap water and sealed via rehydration.

### Detection of glutamic oxaloacetic transaminase (GOT) and glutamic pyruvic transaminase (GPT)

GOT and GPT in blood can be used as indicators for evaluatingliver damage, and we evaluated the hepatotoxicity of erianin in mice using GOT and GPT assays. After blood was taken from the eyeballs of the mice, the blood was incubated overnight at 4 °C and centrifuged at 4 °C (2000 rpm, 15 min). The upper serum was taken for the detection of GOT and GPT. Serum (5 µl) was added to the 96-well plate, 20 µl of preheated matrix solution was added at 37 °C, and the plate was incubated at 37 °C for 30 min. Then, 2,4-dinitrophenylhydrazine solution (20 µl) was added, and the mixture was reacted at 37 °C for 20 min. Finally, 0.4 mol/L sodium hydroxide (200 µl) was added, the 96-well plate was gently shaken at room temperature for 15 min at a wavelength of 510 nm, and the optical density (OD) of each well was determined according to the absolute OD value (determined OD value - control OD value). The corresponding activity units of GOT and GPT were obtained by checking the standard curve.

### Predicting the target genes of erianin in TNBC

The PubChem database (https://pubchem.ncbi.nlm.nih.gov/) contains biological activity data for small organic molecules, and the molecular structure of erianin was obtained from PubChem. The PharmMapper (http://www.lilab-ecust.cn/pharmmapper/) server was designed to identify potential target candidates for a given small molecule. The Comparative Toxicogenomics Database (CTD; http://ctdbase.org/) provides key information about gene‒protein interactions and chemical–disease and gene–disease relationships. The genes related to erianin were screened in the PharmMapper and CTD databases with “erianin” as the keyword. The GeneCards (https://www.genecards.org/) and OMIM (http://www.omim.org/) databases were searched using the keyword “TNBC” and the species “*Homo sapiens*”, respectively. These two sets were subsequently combined, and duplicates were removed using the UniProt (https://www.uniprot.org/) database. Furthermore, Venn diagrams were generated to display the common targets.

### Construction of protein‒protein interactions and hub gene networks

The online network analysis platform STRING 11.5 [24] (http://string-db.org/) was used to analyze the network topology. The common targets were uploaded to the STRING 11.5 database to construct PPIs with a confidence score > 0.9, and the species “*Homo sapiens*” was selected. The PPI network was then visualized and analyzed using Cytoscape 3.8.0. NetworkAnalyzer and CytoNCA were subsequently used to screen the hub genes based on degree centrality in the PPI network.

### Gene expression and survival analysis

The breast cancer gene expression dataset was downloaded from The Cancer Genome Atlas (TCGA) database (https://www.cancer.gov/) after filtering patients according to the following criteria: breast, TCGA, TCGA-BRCA, ductal and lobular neoplasm, and female. Files: transcriptome profiling, gene expression quantification, and HTSeq-FPKM. The dataset comprises samples of tumor tissue and paraneoplastic normal tumors. The female participants were analyzed through transcriptome profiling and gene expression quantification using the HTSeq-FPKM dataset. The samples consisted of both tumor tissues and paraneoplastic normal samples, and patient-related clinical data were also downloaded. TNBC patients were selected based on clinical information screening for PR-negative, ER-negative, and Her-2-negative conditions through either immunohistochemistry or FISH. The expression matrices of both the normal and tumor groups were analyzed using R software. In addition, survival analysis of potential target genes of erianin action in TNBC was performed online using Kaplan‒Meier Plotter (https://kmplot.com/analysis/). The best nodes were selected for grouping, and the ER, PR and Her-2 statuses were set to negative.

### Molecular docking analysis

Molecular docking can be used to predict the binding abilities of small molecular compounds to target genes effectively. The 2D structure of erianin was downloaded from the PubChem database. Chem3D software was subsequently used to convert the 2D structure into a 3D structure. The 3D crystal structures of the 8 hub genes were retrieved from the PDB database. AutoDockTools 1.5.6 software was used to remove all ligands from the protein receptors and add hydrogens and charges before molecular docking simulation. Ligands and protein receptors were recorded in PDBQT format. The binding sites of erianin to the target gene receptor protein were subsequently examined using PyMOL 2.4.1. The binding energy was determined from the affinity. The higher the absolute affinity value is, the stronger the binding affinity of the erianin protein.

### Cell transfection

MDA-MB-231 cells (5 × 10^4^) were seeded in 48-well plates. After 24 h, 100 nM si-SRC and the si-native control (Guangzhou RiboBio Biotechnology Co., Ltd.) were introduced into the cells using TransIntro® EL Transfection Reagent (FT201-01, trans, China) according to the manufacturer’s instructions. After 6 h, the cells were switched to fresh DMEM containing 10% FBS and cultured for 18 h.

### Cellular thermal shift assay (CETSA)

CETSA is an assay that detects the efficiency of intracellular drug binding to target proteins based on the principle that target proteins usually become stable when bound to drug molecules. That is, as the temperature increases, the protein degrades; when the protein binds to the drug, the amount of undegraded protein increases at the same temperature. The CETSA can be used to detect the binding of small molecules to target proteins. MDA-MB-231 cells were harvested and resuspended in PBS containing 0.5% PMSF. Subsequently, the cells were lysed by three repeated freeze‒thaw cycles using liquid nitrogen and a 37 °C water bath. The cell suspension was centrifuged (12,000 rpm, 4 °C, 20 min), after which the supernatant was collected and divided into two groups. One group was treated with erianin, and the other group was treated with the same amount of DMSO for 30 min at 37 °C. Then, the samples were incubated at various temperatures (50 °C, 55 °C, 60 °C, 65 °C) for 3 min, cooled to room temperature for an additional 3 min, and finally centrifuged (12,000 rpm, 4 °C, 20 min). The protein content in the supernatant was collected and analyzed via WB.

### RNA sequencing

Total RNA was extracted using TRIzol Reagent (Novogene Technology Co., Ltd.). High-throughput sequencing and analyses were subsequently performed on the mRNAs. The integrity of the RNA was tested on an Agilent 2100 bioanalyzer according to the manufacturer’s instructions, and the library was constructed with the NEBNext® Ultra™ Directional RNA Library Prep Kit for Illumina®. After the library was constructed, the library was initially quantified using a Qubit2.0 fluorometer, and the library was diluted to 1.5 ng/µl. The library’s insert size and effective concentration were subsequently detected by an Agilent 2100 bioanalyzer and qRT‒PCR. Finally, the total RNA from the indicated cells was subjected to mRNA sequencing on an Illumina NovaSeq 6000 platform. DESeq2 was used to identify the differentially expressed genes (DEGs). ClusterProfiler software was used to carry out a GO functional enrichment analysis and a KEGG pathway enrichment analysis of the differential gene sets.

### Determination of cholesterol levels

A total cholesterol assay kit was used to detect the effects of different drug treatments on cholesterol levels in TNBC cells or tumor tissues. Cell samples: The prepared cell suspension was centrifuged (1500 rpm, 4 °C, 5 min), the supernatant was discarded, and the cell residue was left. RIPA lysis buffer was added for homogenization, and the cell sample was lysed by ultrasonication in an ice water bath. For tissue samples, RIPA lysis buffer and steel balls were added to the tissue block, which was subsequently ground in a tissue grinder. The homogenate prepared from the two samples was not centrifuged. During cholesterol detection, 200 µl of the working solution was added to a 96-well plate, after which 5 µl of the homogenate or standard solution was added. Following mixing, the homogenate was incubated at 37 °C for 10 min. The absorbance of each sample was measured at a wavelength of 510 nm. In addition, 100 µl of the homogenate was centrifuged (12,000 rpm, 4 °C, 30 min), and BCA was used to determine the protein concentration. Cholesterol levels were calculated as [(A sample-A blank)/(A standard-A blank)×C standard]/Cpc (C standard: standard concentration, Cpr: homogenate protein concentration).

### Western blotting (WB)

TNBC cells were cultured at a density of 1 × 10^6^ per well in 6-well plates. After treatment with 40 nM erianin for 24 h, the cells were lysed in precooled RIPA buffer. RIPA lysis buffer and steel balls were added to the tumor tissue blocks, which were ground in a tissue grinder. A BCA protein assay kit was used to determine the protein concentrations. Equal amounts of protein were separated by SDS‒PAGE and transferred to 0.45 μm polyvinylidene fluoride membranes (Millipore, IPVH00010)1. After blocking with 2% BSA for 2 h at room temperature, the membranes were incubated with primary antibodies at 4 °C overnight. Then, the membranes were washed with TBST buffer 3 times and incubated with an HRP-conjugated secondary antibody. The results were developed and recorded by a chemiluminescent analysis system (Tanon-5200, Tanon Science and Technology). ImageJ software was used for data analysis.

### Immunohistochemistry (IHC)

The tumor tissues were fixed in 4% paraformaldehyde for 24 h, washed in running water, and embedded in paraffin. Then, the paraffin-embedded tissue was cut into 5 μm thick sections. Sodium citrate buffer was used to boil the slides for 10 min to retrieve the antigens. After blocking with goat serum solution for 30 min, the sections were incubated overnight at 4 °C with primary antibodies. The next day, a secondary antibody was introduced, followed by the addition of DAB chromogenic reagent and counterstaining with hematoxylin. The tissue sections were scored based on the intensity of the immunohistochemical signal and the number of areas that were positively stained. Finally, Image-Pro Plus 6.0 was used for quantitative analysis of the images.

### Real-time PCR

TRIzol reagent (Invitrogen) was used to extract total RNA from frozen cell samples. The RNA quality and concentration were determined via agarose gel electrophoresis and spectrophotometry. Single-stranded complementary DNA was obtained by reverse transcription of 1 µg of RNA using an RT‒PCR kit (BD Biosciences, Franklin, NJ, USA). The mRNA levels of the genes detected were quantified using an Applied Biosystems 7300 Real-Time PCR System (USA) with SYBR Green Master Mix (Takara Bio, Osaka Prefecture, Osaka City, Japan). Gene expression was analyzed using the 2^−ΔΔCt^ method. The primers used for the real-time PCR analysis of genes related to cholesterol synthesis and uptake are shown in Table 1. Each experiment had three independent replicates.

### Statistical analysis

All the values are expressed as the means ± SDs. All the statistical analyses and graphs were generated using GraphPad Prism software. Significant differences between mean values were determined by ANOVA, followed by Fisher’s protected least significance difference test.

## Results

### Erianin exerts anti-TNBC effects in vitro

**Fig. 1:**
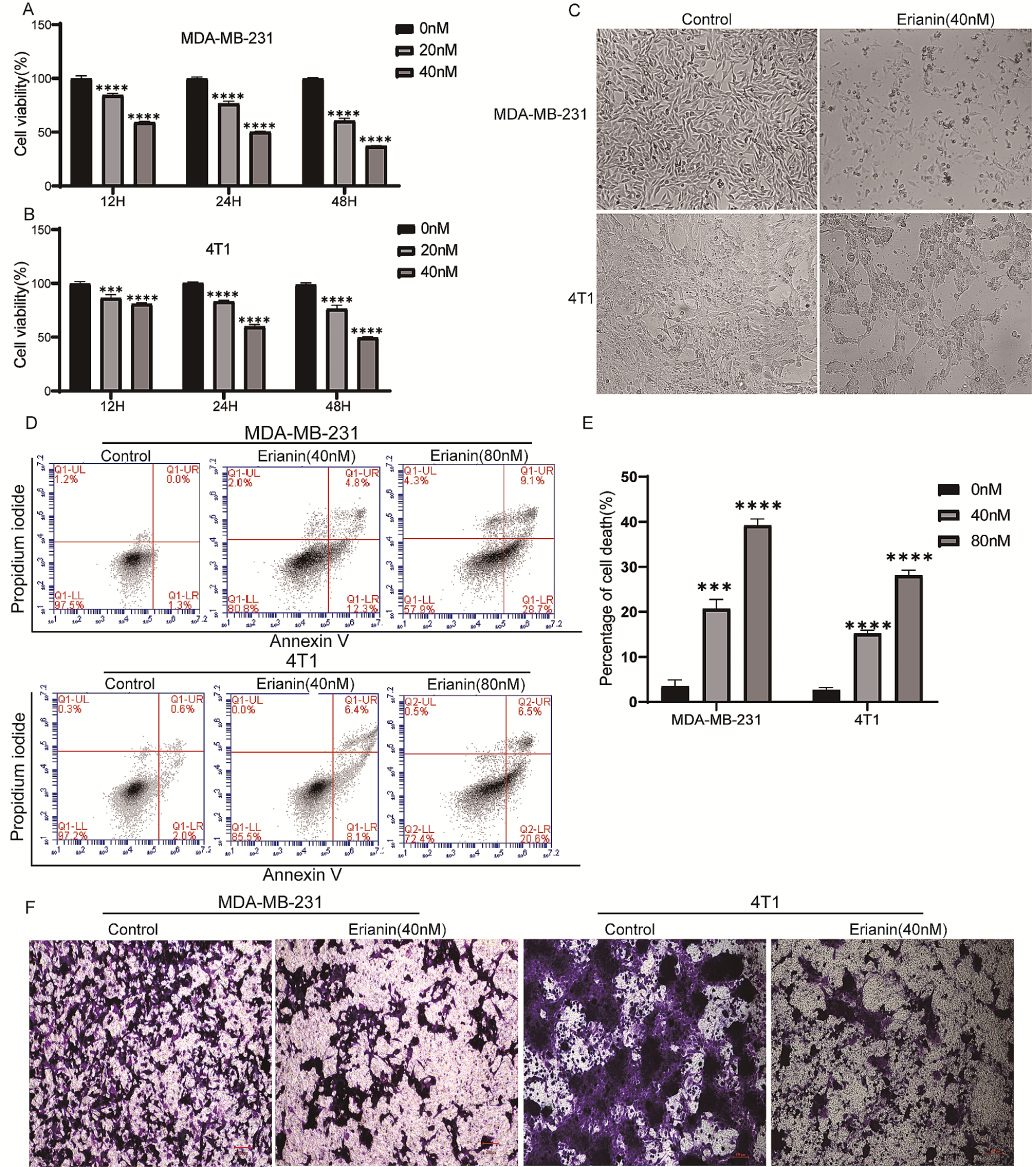
Erianin inhibits the progression of TNBC cells in vitro. MDA-MB-21 (**A**) and 4T1 (**B**) cells were treated with different concentrations (0, 20 and 40 nM) of erianin for different times (12, 24 and 48 h), after which cell viability was determined via CCK-8 assay. **C**. Changes in the morphology of TNBC cells were examined before and after exposure to 40 nM erianin. **D**. Flow cytometry results showing the distribution of MDA-MB-231 and 4T1 cell apoptosis after treatment with erianin for 24 h. **E**. Statistical analysis of apoptosis. **F**. Transwell assays were used to evaluate the effect of erianin treatment on the migration of TNBC cells. The data are presented as the means ± SDs (*n* = 3); ***p < 0.001, ****p < 0.0001 compared to the control groups

To evaluate the effect of erianin on the proliferation of TNBC cells, MDA-MB-231 and 4T1 cells were treated with DMSO or different concentrations (0, 20 and 40 nM) of erianin for different times (12, 24 and 48 h). Then, a CCK-8 assay was used to detect cell proliferation. The results showed that there was a time- and dose-dependent inhibition of erianin on TNBC cell proliferation (Fig. 1A, B). Moreover, after treatment with erianin for 24 h, erianin induced morphological changes, including cell shrinkage, an increase in the number of floating cells and increased cell debris in MDA-MB-231 and 4T1 cells (Fig. 1C). Next, we explored the roles of erianin in cell apoptosis and migration. According to the flow cytometry analysis, erianin considerably induced TNBC cell apoptosis (Fig. 1D, E). Transwell assays also showed that erianin significantly inhibited TNBC cell migration (Fig. 1F). Taken together, these results indicated that erianin can inhibit the development of TNBC cells in vitro.

### Erianin inhibits the growth of transplanted tumors in vivo

**Fig. 2:**
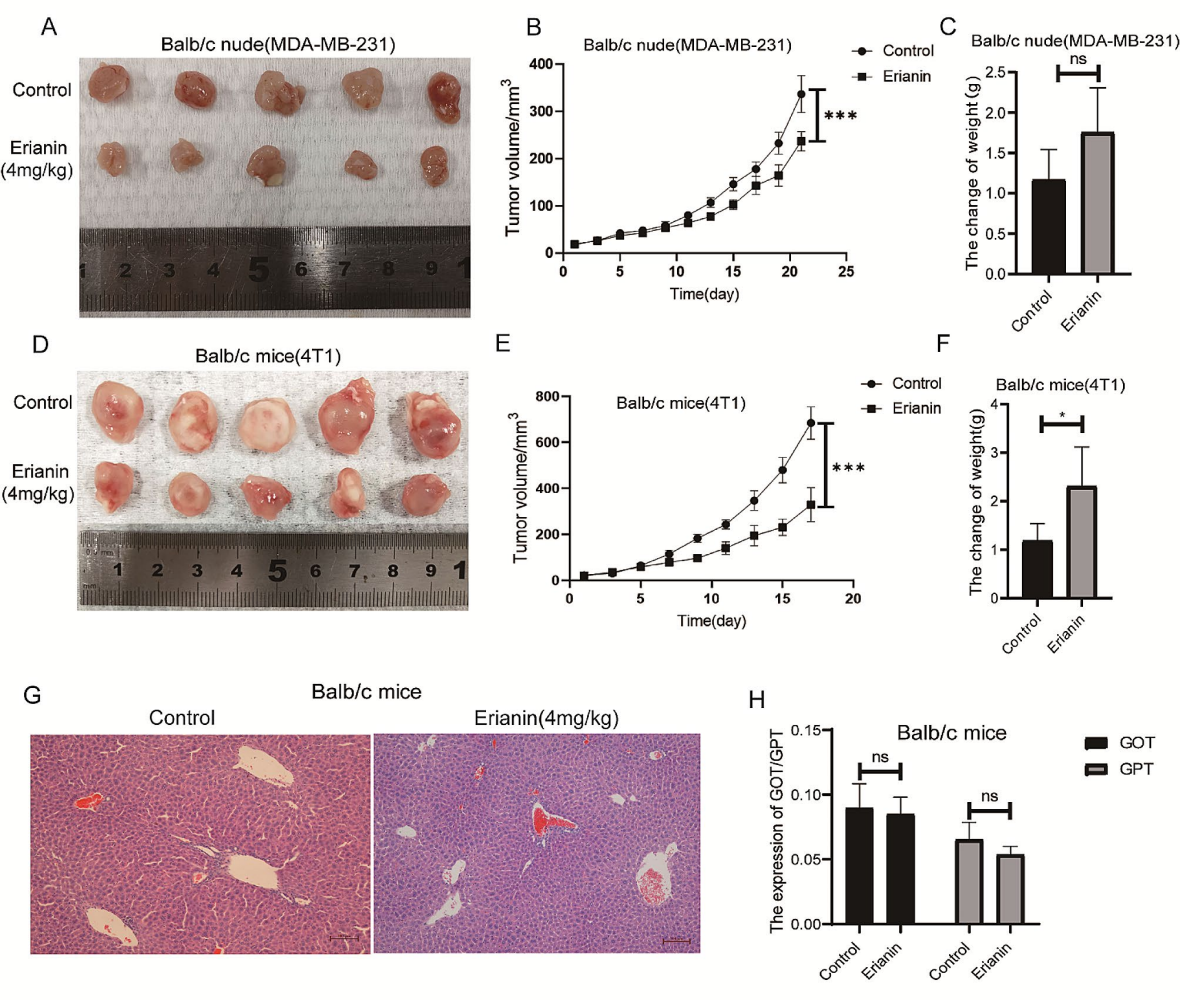
Erianin inhibits the proliferation of TNBC cells *in vivo.***A**. A representative image of the xenograft tumors at the end of the experiment. **B**. Tumor volumes in the different groups. **C**. Changes in the body weight of BALB/c nude mice in each group. **D**. A representative image of the xenograft tumors at the end of the experiment. **E**. Tumor volumes in the different groups. **F**. Changes in the body weight of BALB/c mice in each group. **G**. Effect of erianin on liver injury in BALB/c mice. **H**. Effect of erianin on the blood content of GOT and GPT. The data are presented as the means ± SDs (*n* = 3); *p < 0.05, ***p < 0.001, ****p < 0.0001 compared to the control group

To investigate the antitumor effect of erianin in vivo, MDA-MB-231 cells and 4T1 cells were injected into BALB/c nude mice and BALB/c normal mice in situ. When the tumor size reached approximately 15 mm^3^, the mice were randomized into control (DMSO) or erianin (4 mg/kg) groups. Consistent with the in vitro results, erianin treatment obviously suppressed the growth of tumors (Fig. 2A, B and D, E). While, after the administration of erianin, the weight of the mice did not decrease (Fig. 2C and F). Further HE staining revealed no significant difference in liver morphology between the erianin group and the control group (Fig. 2G). Moreover, erianin did not cause an increase in the blood concentration of GOT or GPT (Fig. 2H). Collectively, these results demonstrated that erianin obviously inhibited tumor growth and had no liver toxicity in vivo.

### SRC is a potential target of erianin in TNBC

**Fig. 3:**
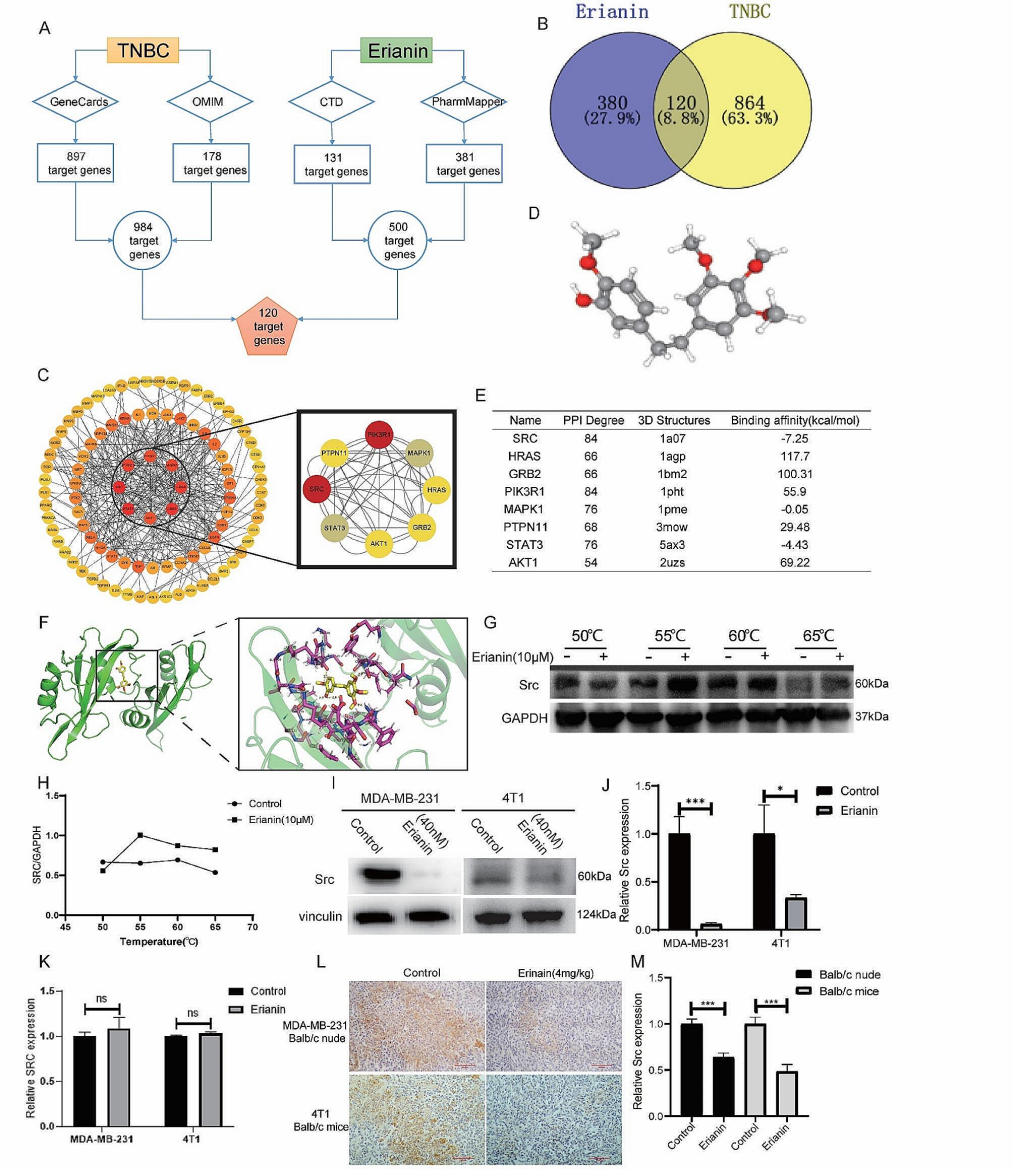
Erianin inhibits the expression of SRC in TNBC. **A**. Schematic diagram of 120 target genes. **B**. Venn diagram of common target genes between erianin and TNBC. **C**. PPI network of the hub genes. **D**. The 3D structure of erianin. **E**. Molecular docking results of erianin with target proteins. **F**. Molecular docking visualization results showing that erianin binds to SRC. **G**. The binding capacity of erianin with SRC was measured through CETSA. **H**. Quantitative analysis of the relative expression levels of SRC in CETSA. **I**. After 24 h of treatment with erianin, the expression of SRC was analyzed via WB. **J**. Quantitative analysis of the relative expression levels of SRC by Western blotting. **K**. After 24 h of treatment with erianin, the expression of SRC was analyzed via RT-qPCR. **L**. Representative images of IHC staining for SRC in xenograft tumors after treatment with erianin. **M**. Quantitative analysis of the relative expression levels of SRC by IHC. The data are representative of 3 independent experiments and are presented as the mean ± standard deviation. *p < 0.05, **p < 0.01, ***p < 0.001

Based on network pharmacology, 381 and 131 genes related to erianin were obtained from the PharmMapper and CTD databases, respectively. A total of 178 TNBC-related genes were identified by searching the OMIM database, and 897 TNBC-related genes were identified in the GeneCards database. After removing duplicate genes, 984 genes were selected for further analysis. Thereafter, both erianin targets and TNBC related genes were analyzed via a Venn diagram, and 120 common targets were identified (Fig. 3A, B).

To further explore the targets of erianin, the above 120 common targets were uploaded to the STRING database to construct the PPI network. Cytoscape software was used to visualize the PPI network, and 120 nodes and 600 edges were obtained (Fig. 3C). To identify the core target genes, node degree was utilized as a screening criterion, and the top 8 core target genes were selected: SRC, STAT3, AKT1, GRB2, HRAS, MAPK1, PIK3R1 and PTPN11. Subsequently, we obtained the 3D structure of erianin to predict its reliable target (Fig. 3D). AutoDock was used to calculate the binding active pockets and binding energies between erianin and the 8 hub genes. The smaller the binding energy is, the stronger the binding force and the more stable the structure. As shown in Fig. 3E, erianin had the strongest binding with SRC, and its potential interaction is shown in Fig. 3F. These data suggested that erianin may affect the progression of TNBC by interacting with SRC.

To further verify the interaction of erianin with SRC, we conducted a CETSA to determine the binding capacity of erianin for SRC. MDA-MB-231 cells were exposed to 10 µM erianin at different temperatures. The results showed that the stability of SRC was significantly enhanced by binding with erianin, indicating that SRC could be a potential protein target of erianin (Fig. 3G, H). Next, we examined the effect of erianin on SRC expression. MDA-MB-231 and 4T1 cells were treated with erianin for 24 h. The results showed that the expression of the SRC protein was significantly inhibited by erianin (Fig. 3I, J). However, Erianin did not have a significant effect on SRC gene expression (Fig. 3K). Moreover, in two xenograft transplantation models, the expression of SRC was also decreased in the erianin group (Fig. 3L, M), which suggested that erianin can inhibit the expression of SRC in TNBC.

### SRC can affect TNBC cell proliferation in vitro and in vivo

**Fig. 4:**
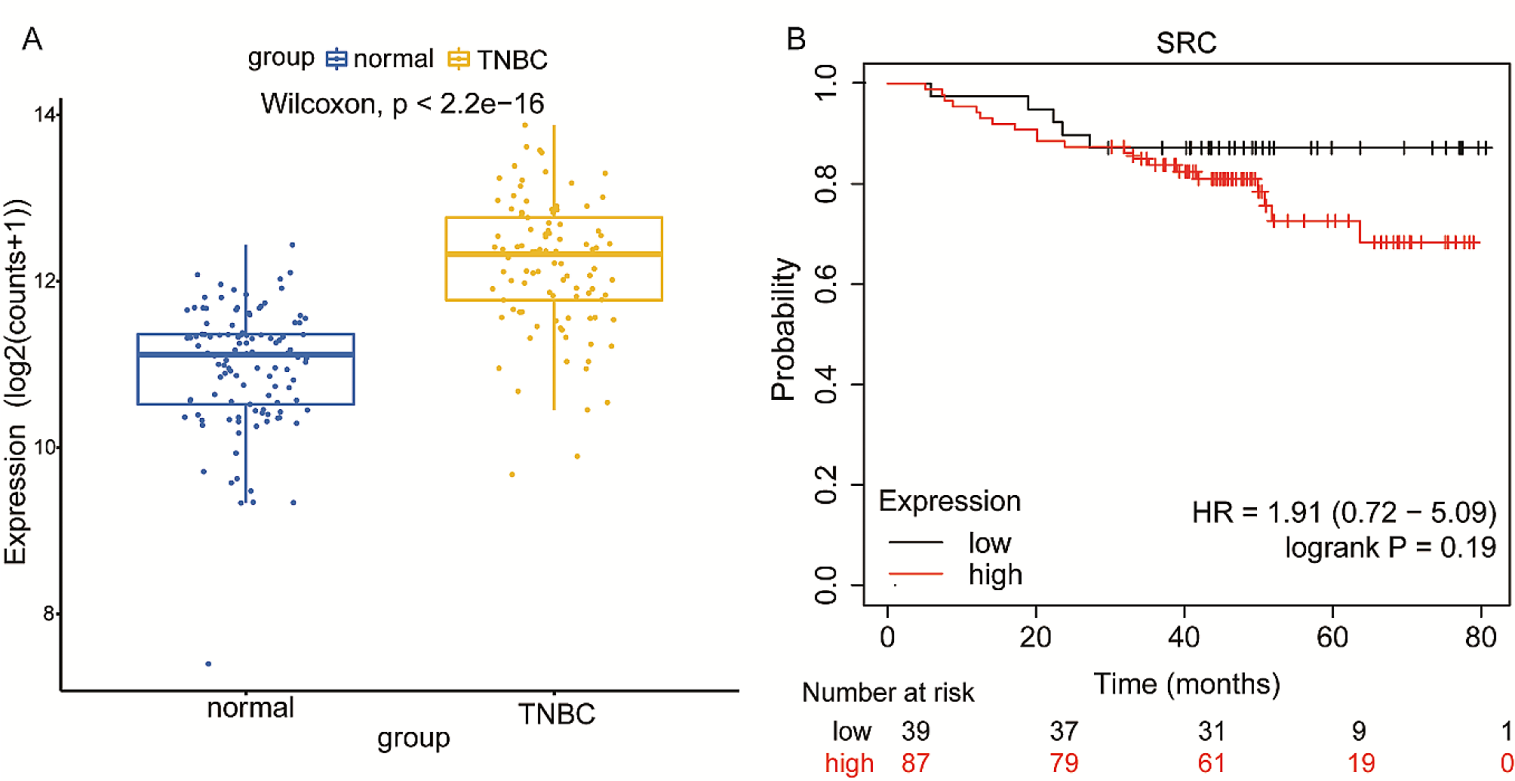
Expression of SRC in TNBC patients and its effect on survival. **A**. The expression of SRC in TNBC tissues was significantly greater than that in normal tissues. **B**. Effect of SRC expression on survival in TNBC patients

**Fig. 5:**
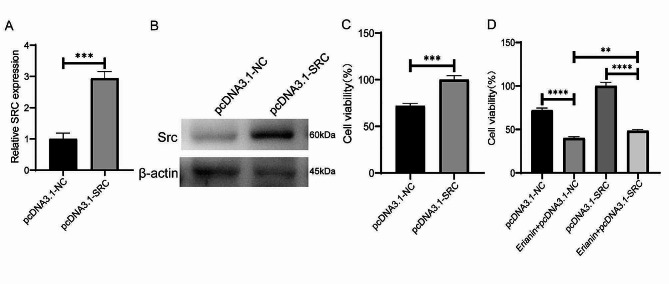
Overexpression of SRC in MDA-MB-231 cells promoted cell proliferation and attenuated the inhibitory effect of erianin on cell proliferation. **A**. The expression of SRC gene in MDA-MB-231 cells after the overexpression of SRC. **B**. The expression of Src protein in MDA-MB-231 cells after the overexpression of SRC. **C**. Effect of SRC overexpression on the proliferation of MDA-MB-231 cells. **D**. SRC overexpression attenuated the inhibitory effect of erianin on the proliferation of MDA-MB-231 cells. The data are presented as the means ± SDs (*n* = 3); **p < 0.01, ***p < 0.001, ****p < 0.0001 compared to the control group

**Fig. 6:**
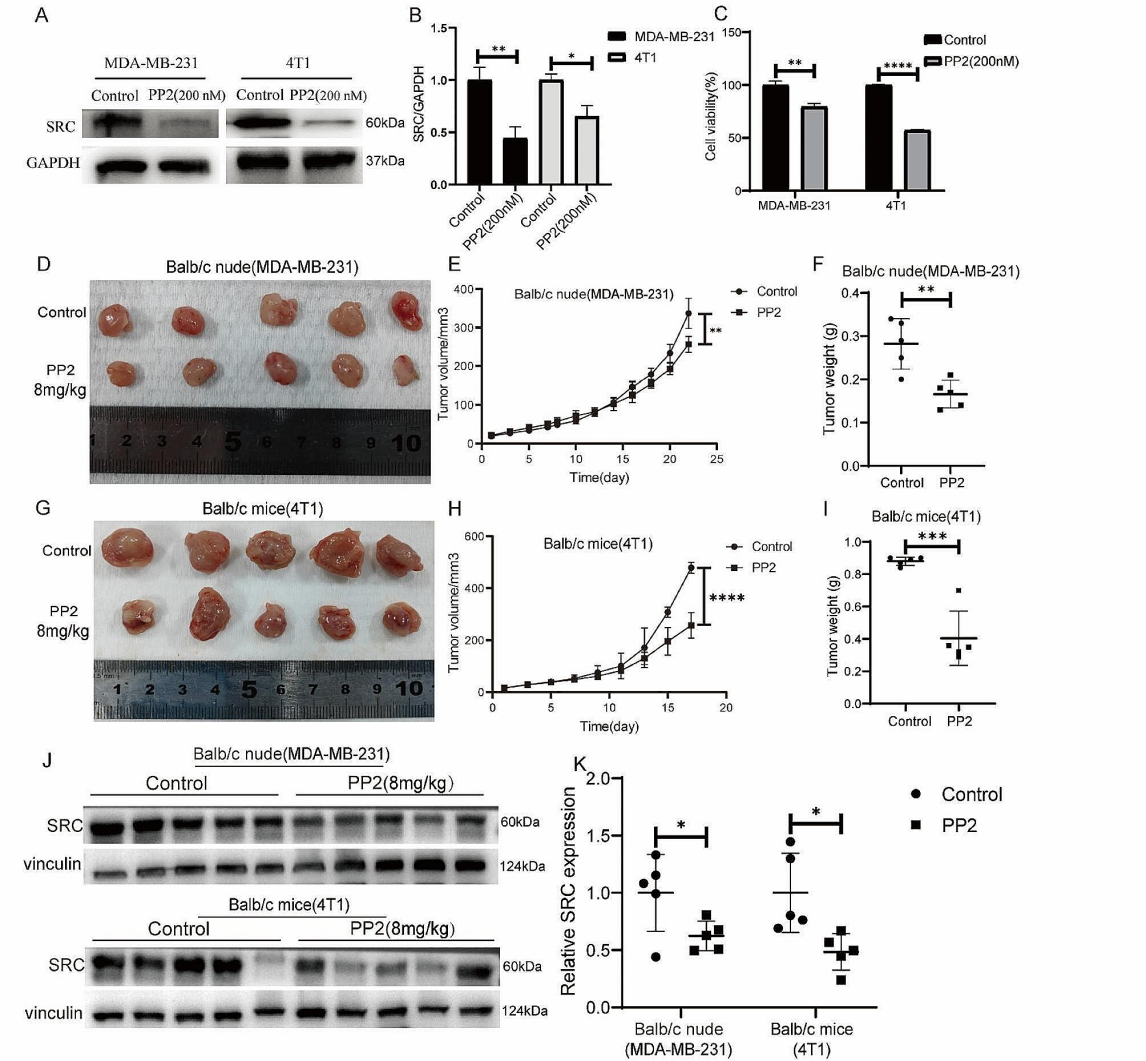
Low SRC expression inhibits the proliferation of TNBC cells in vitro and in vivo. **A**. Changes in Src protein levels in MDA-MB-231 and 4T1 cells treated with PP2. **B**. Statistical analysis of the changes in SRC protein levels in two TNBC cell lines treated with PP2. **C**. CCK-8 assay of MDA-MB-231 and 4T1 cells treated with PP2. **D**. Images of BALB/c nude mouse xenografts after PP2 treatment. **E**. Tumor volume in each group (*n* = 5). **F**. Tumor weight in each group (*n* = 5). **G**. Images of BALB/c mouse xenografts after PP2 treatment. **H**. Tumor volume in each group (*n* = 5). **I**. Tumor weight in each group (*n* = 5). **J**. The expression of SRC in two kinds of transplanted tumors treated with PP2. **K**. Quantitative analysis of protein expression in J. The data are presented as the means ± SDs (*n* = 3); *p < 0.05, **p < 0.01, ***p < 0.001, ****p < 0.0001 compared to the control groups

The above results suggested that the anticancer effect of erianin may be influenced by SRC in TNBC. Subsequently, we obtained a total of 101 TNBC samples and 113 normal tissue samples by screening TCGA database analysis, and the expression of SRC in TNBC tissues was significantly greater than that in normal tissue samples (Fig. 4A). Survival analysis via Kaplan‒Meier Plotter showed that TNBC patients with high SRC expression had a relatively poor prognosis, although the results were not significantly different (*p* > 0.05) (Fig. 4B). To investigate the role of SRC in TNBC cell proliferation, we overexpressed the SRC gene in MDA-MB-231 cells (Fig. 5A, B), and CCK-8 analysis showed that the SRC-overexpressing cells exhibited greater proliferation than the control cells did (Fig. 5C). Moreover, we found that SRC overexpression attenuated the inhibitory effect of erianin on the proliferation of MDA-MB-231 cells (Fig. 5D). Then, we further treated MDA-MB-231 and 4T1 cells with PP2, a specific inhibitor of SRC. The results showed that PP2 significantly reduced the expression of the SRC protein in cells (Fig. 6A, B), and the proliferation of cells with low SRC expression was significantly inhibited (Fig. 6C). These results suggested that erianin may affect TNBC proliferation by downregulating SRC. To further confirm these findings, we established two xenograft transplantation models, and the results showed that, compared with those in the control group, the tumor volume and weight were significantly lower in the PP2 treatment group (Fig. 6D-I), as was the level of SRC expression in tumor tissues (Fig. 6J, K). These results were consistent with the in vitro findings, indicating that SRC plays a vital role in the antitumor effects of erianin.

### Erianin inhibits the proliferation of TNBC by reducing cholesterol levels

**Fig. 7:**
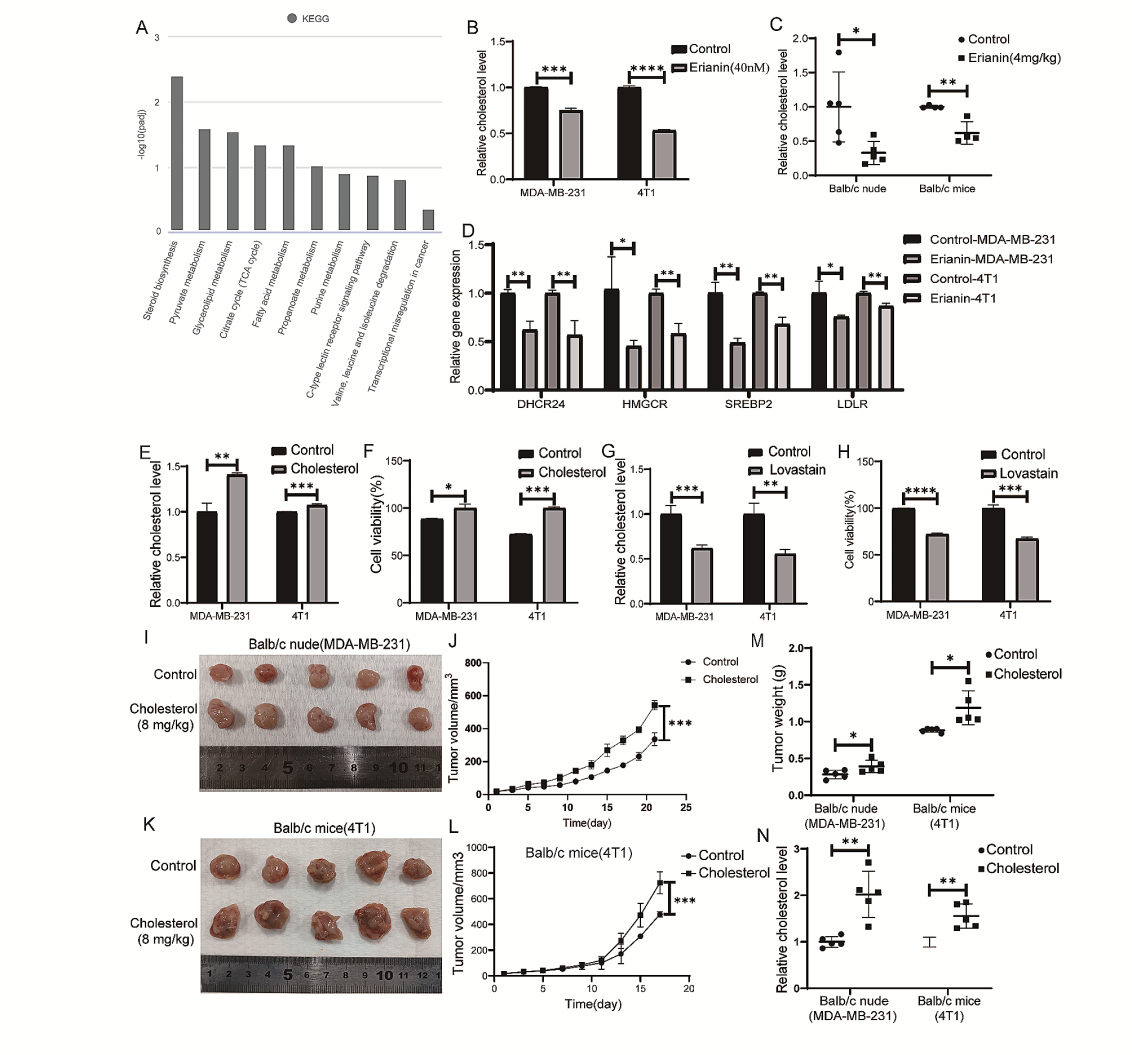
Erianin inhibits TNBC cell proliferation in vitro and in vivo by reducing cholesterol levels. **A**. KEGG pathway analysis. **B**. Changes in cholesterol levels in TNBC cells after treatment with erianin. **C**. Changes in cholesterol levels in transplanted tumors from mice after treatment with erianin. **D**. RT-PCR analysis of HMGCR, SERBP2, DHCR24, and LDLR in erianin-treated cells. **E**. Changes in intracellular cholesterol levels in TNBC cells treated with cholesterol. **F**. CCK-8 assay of TNBC cells treated with cholesterol for 24 h. **G**. Changes in intracellular cholesterol levels in TNBC cells treated with lovastatin. **H**. CCK-8 assay of TNBC cells treated with lovastatin. **I**. MDA-MB-231 cells were injected into BALB/c nude mice in situ, and images of xenograft tumors after cholesterol treatment were shown. **J**. Tumor volume in each group (*n* = 5). **K**. 4T1 cells were injected into BALB/c mice in situ, and images of xenograft tumors after cholesterol treatment were shown. **L**. Tumor volume in each group (*n* = 5). **M**. Tumor weight in each group (*n* = 5). **N**. Cholesterol levels in tumors from two kinds of transplanted tumors. The data are presented as the means ± SDs (*n* = 3); *p < 0.05, **p < 0.01, ***p < 0.001, ****p < 0.0001 compared to the control group

To gain insight into the signaling pathway underlying the erianin-mediated inhibition of cell proliferation, transcriptome analysis was performed. KEGG pathway analysis indicated that the steroid biosynthesis pathway was the most downregulated pathway in the erianin group (Fig. 7A; Table 2), and cholesterol was the most common class of steroids. Thus, we confirmed these findings by measuring cellular cholesterol levels and found that erianin significantly decreased the cholesterol levels in MDA-MB-231 cells and 4T1 cells (Fig. 7B). The same results were observed in transplanted tumors in mice (Fig. 7C). Further analysis revealed that the expression of four molecules involved in cholesterol synthesis and absorption, HMGCR, SERBP2, DHCR24 and LDLR, was significantly inhibited upon erianin treatment (Fig. 7D). The above results indicated that erianin can reduce cholesterol levels in TNBC patients. Studies have shown that cholesterol can promote the proliferation of tumor cells. To investigate the effect of cholesterol on the proliferation of TNBC cells, we treated MDA-MB-231 and 4T1 cells with cholesterol and lovastatin (an inhibitor of cholesterol synthesis), respectively. The results showed that cholesterol increased the cholesterol level in the two kinds of TNBC cells (Fig. 7E) and significantly promoted cell proliferation (Fig. 7F). In contrast, lovastatin reduced the cholesterol level in both TNBC cell lines (Fig. 7G) and significantly suppressed cell proliferation (Fig. 7H). Two mouse orthotopic transplanted tumor models were constructed to investigate the impact of cholesterol on tumor growth in vivo. The results showed that the tumor growth rate, tumor volume and weight in the cholesterol group were significantly greater than those in the control group (Fig. 7I-M). Moreover, the cholesterol level in the tumors of the cholesterol group was significantly increased (Fig. 7N).

**Fig. 8:**
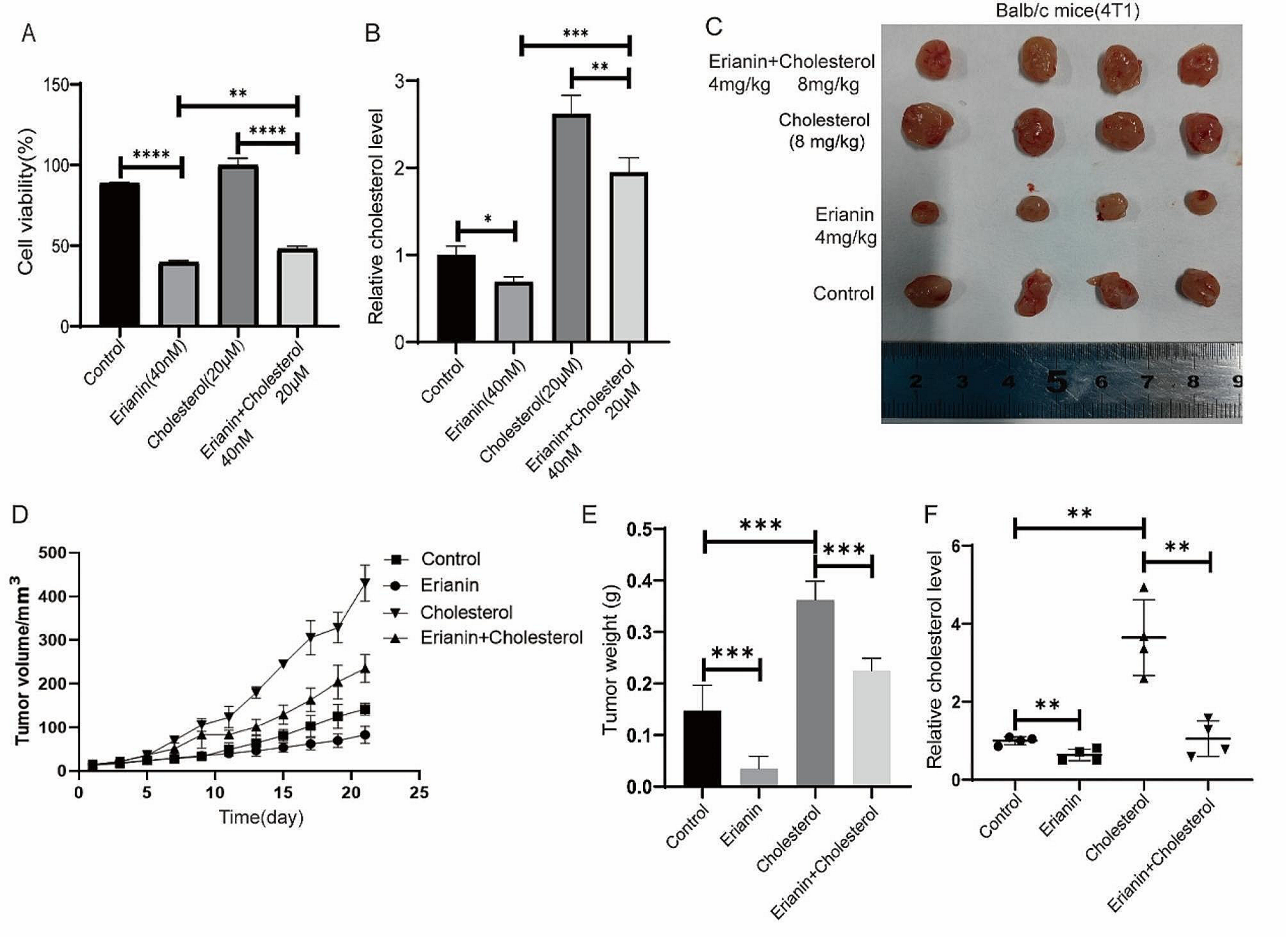
High cholesterol rescues the inhibitory effect of erianin on TNBC proliferation. **A**. Cell proliferation detection after MDA-MB-231 cells were treated with erianin and cholesterol. **B**. Changes in cholesterol levels after treatment with erianin and cholesterol in MDA-MB-231 cells. **C**. Representative image of xenograft tumors at the endpoint of the experiment (*n* = 4). **D**. The tumor volume in the different groups (*n* = 4). **E**. Tumor weight in each group (*n* = 4). **F**. Cholesterol levels in transplanted tumors. *p < 0.05, **p < 0.01, ***p < 0.001, ****p < 0.0001 compared to the control groups

Subsequently, we treated MDA-MB-231 cells with both 40 nM erianin and 20 µM cholesterol for 24 h and found that the decreases in cell proliferation and cholesterol levels induced by erianin were partially rescued by exogenous cholesterol treatment (Fig. 8A, B). These results were further confirmed in vivo. Cholesterol treatment interfered with the inhibitory effect of erianin on tumor growth and restored cholesterol levels (Fig. 8C-F). Taken together, these results indicated that erianin inhibits TNBC cells proliferation by reducing cholesterol levels in vitro and in vivo.

### Erianin inhibits cholesterol metabolism by downregulating SRC and affects the proliferation of TNBC cells

**Fig. 9:**
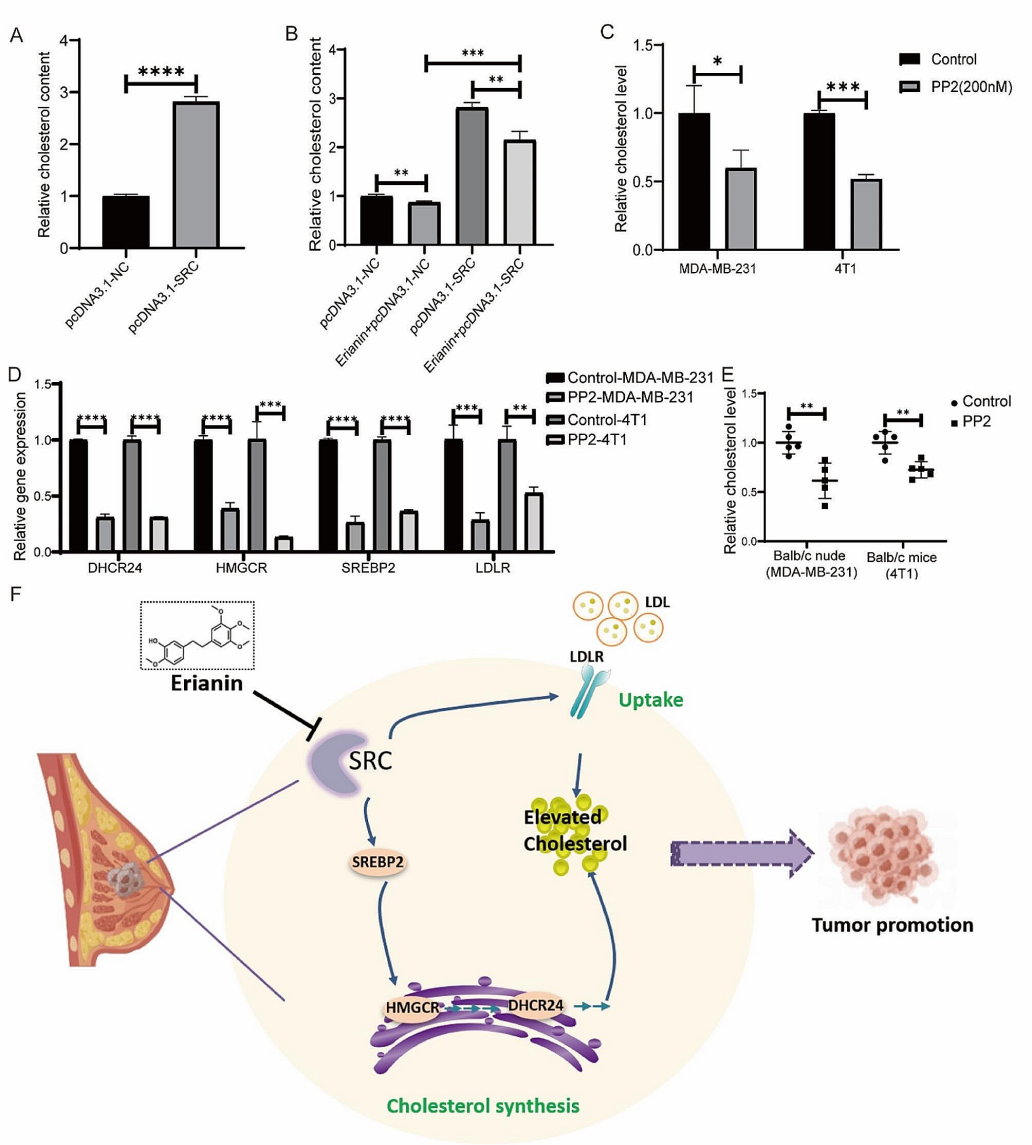
Erianin inhibits the progression of TNBC by suppressing SRC-mediated cholesterol metabolism. **A**. Changes in the intracellular cholesterol concentration in MDA-MB-231 cells after the overexpression of SRC. **B**. Changes in cholesterol content after the overexpression of SRC in MDA-MB-231 cells treated with erianin. **C**. PP2 decreased the cholesterol levels in TNBC cells. **D**. RT-PCR analysis of HMGCR, SERBP2, DHCR24, and LDLR in PP2-treated cells. **E**. PP2 reduced cholesterol levels in mouse xenograft tumors. **F**. Molecular mechanism by which erianin inhibits TNBC progression. The data are presented as the means ± SDs (*n* = 3); *p < 0.05, **p < 0.01, ***p < 0.001, ****p < 0.0001 compared to the control group

Our study suggested that erianin may regulate TNBC cell proliferation by targeting the SRC molecule and that it can also regulate the cholesterol metabolism pathway. We then investigated whether erianin decreases cholesterol levels via SRC. To determine the relationship between SRC and cholesterol levels in TNBC, we first overexpressed SRC in MDA-MB-231 cells and showed that the overexpression of SRC significantly increased cholesterol levels in the cells (Fig. 9A). Moreover, SRC overexpression rescued the inhibitory effect of erianin on cholesterol (Fig. 9B). We then treated TNBC cells with 200 nM PP2. After 24 h, compared with those in the control group, the cholesterol levels in the MDA-MB-231 and 4T1 cells were significantly lower (Fig. 9C), and the expression of genes related to cholesterol synthesis and absorption was significantly lower (Fig. 9D). Moreover, PP2 treatment significantly reduced the cholesterol level in vivo (Fig. 9E). These results demonstrated that erianin inhibits the progression of TNBC by downregulating SRC-mediated cholesterol metabolism (Fig. 9F).

## Discussion

Erianin is an active compound found in Dendrobium that has various effects, including antioxidant, antiangiogenic, and antitumor effects. Earlier investigations have suggested that erianin may regulate the onset and progression of several cancer types, including lung and liver cancer [16, 25], by suppressing cell proliferation, inducing cell cycle arrest, and enhancing apoptosis. Limited research has revealed that erianin can inhibit the proliferation and migration of the BC cell line T47D and induce cell apoptosis [21]. However, further in vivo experiments have not been conducted, and the drug targets and specific molecular mechanisms of erianin in TNBC have not been elucidated.

This study aimed to elucidate the regulatory role of erianin in TNBC progression and identify its drug target and molecular mechanism. We found that erianin obviously inhibit the proliferation and migration of TNBC cells and the growth of transplanted tumors, meanwhile it had no liver toxicity in vivo. To investigate the mechanism of erianin in TNBC, we performed network pharmacology analysis. The analysis revealed 120 potential target genes shared by erianin and TNBC. To determine the crucial targets of erianin in the anti-TNBC process, we conducted a PPI analysis, the results revealed that SRC, STAT3, AKT1, GRB2, HTRAS, MAPK1, PIK3R1, and PTPN11 were eight significant hub proteins.

Notably, these eight genes are commonly associated with the incidence and progression of TNBC in the literature. For instance, various studies had demonstrated that the SRC pathway was a crucial pathway that drives TNBC [26]. SRC-mediated cellular signaling can affect the proliferation, survival, migration, and invasion of TNBC cells [27]. In TNBC, suppression of STAT3 expression inhibited tumor proliferation and migration [28]. Additionally, HRAS [29] and MAPK1 [30] levels were significantly increased in TNBC patients. Similarly, PIK3R1 [31] and AKT1 [32] played crucial roles in the development of TNBC. PTPN11 downregulation may inhibite the proliferation, migration, and invasion of TNBC cells and played a pivotal role in TNBC progression [33].

To further clarify the specific targets of erianin in anti-TNBC, we performed molecular docking experiments. The results indicated that there was a strong binding interaction between erianin and SRC. We then verified the binding efficiency of erianin to SRC proteins by CETSA. SRC is a nonreceptor tyrosine kinase and the first confirmed oncogene. It was also the first protein tyrosine kinase to be described. The SRC functions as a signal transduction center and allows for coordinated cell responses to extracellular stimuli. SRC-mediated abnormal activation of SRC kinase could promote tumor cell movement, proliferation, invasion, and metastasis [34]. So, targeting SRC kinase is an attractive strategy for treating cancer. To investigate the effect of SRC on TNBC proliferation, we inhibited SRC expression with the SRC inhibitor PP2 in two TNBC cell lines. Consequently, SRC expression and cell proliferation ability were significantly reduced. In addition, PP2 also substantially inhibited the growth of transplanted tumors in mice.

To gain a better understanding of the signaling pathways through which erianin inhibited the progression of TNBC, we performed transcriptome sequencing on tumor tissues from mice treated with erianin. The results indicated that erianin strongly inhibited the biosynthesis of steroids, with cholesterol being the largest class of steroids. Cholesterol metabolism is abnormally active in tumor cells, accompanied by elevated levels of intracellular cholesterol and its metabolic products, leading to enhanced proliferation and invasion [35] and tumor drug resistance [36, 37], affecting cell stemness [38], patient survival [39] and recurrence [40]. A large number of retrospective studies have shown that high plasma cholesterol levels are associated with a high risk of BC and are an independent risk factor for BC onset and recurrence [41]. For example, after menopause in women, a high-cholesterol diet increased the risk of BC [42]; compared with mice on a normal diet, mice on a high-fat diet had significantly elevated blood cholesterol levels and accelerated tumor growth [43]. In addition, Qiu T and others observed increased cholesterol biosynthesis in tumor stem cells derived from BC patients, and cholesterol promoted the occurrence of BC by enhancing Hedgehog signaling and tumor stem cell-like cell populations [44]. These studies indicated that cholesterol played a crucial role in the progression of BC. We treated MDA-MB-231 and 4T1 cells with cholesterol and cholesterol synthesis inhibitors (lovastatin). The results indicated that the addition of cholesterol promoted the proliferation of MDA-MB-231 and 4T1 cells. After cholesterol levels were reduced, the proliferation of TNBC cells was inhibited. Moreover, in the mouse-transplanted tumors, cholesterol significantly promoted tumor growth. In addition, we found that erianin significantly reduced cholesterol levels in both TNBC cells and xenografts. RT‒PCR experiments showed that by inhibiting the expression of genes involved in cholesterol synthesis and absorption, erianin can reduce cholesterol levels in TNBC cells. These findings indicated that erianin could inhibit the development of TNBC by reducing cholesterol levels.

Studies have shown that SRC may regulate intracellular cholesterol accumulation through multiple pathways [45–47]. For example, in glioblastoma, SRC could promote cholesterol imbalance through the ERK pathway [48]. Doxorubicin can reduce the expression of the key enzyme HMGCR in cholesterol synthesis by inhibiting the EGFR/SRC pathway [49]. To explore the effect of SRC on cholesterol in TNBC, we overexpressed SRC in MDA-MB-231 cells and found that the intracellular cholesterol levels significantly increased. Treatment of TNBC with PP2 resulted in a significant decrease in intracellular cholesterol levels. Moreover, RT‒PCR showed that PP2 could reduce cholesterol levels in TNBC cells by inhibiting the expression of genes related to cholesterol synthesis and absorption. In addition, we conducted a rescue experiment by treating MDA-MB-231 cells with erianin and overexpressing the SRC gene. The results showed that overexpression of the SRC gene could rescue the inhibitory effect of erianin on MDA-MB-231 cell proliferation and cholesterol. Furthermore, MDA-MB-231 cells were simultaneously treated with erianin and cholesterol. The results showed that high cholesterol levels in MDA-MB-231 cells could also rescue the inhibitory effect of erianin on cell proliferation.

However, this study still has several limitations. For instance, we only used molecular docking and thermal drift experiments to identify erianin targets, and we should further validate the above results using the SM pull-down technique. Moreover, the specific mechanism by which SRC regulates cholesterol metabolism in TNBC needs to be elucidated. Additionally, in future studies, we should investigate SRC expression and cholesterol levels and their correlation with various pathological indicators as well as TNBC patient outcomes in clinical samples.

In conclusion, our study clarified the regulatory role of erianin in the progression of TNBC and identified the drug targets and molecular mechanisms of erianin in the anti-TNBC process. The results showed that erianin significantly reduced TNBC cell proliferation, migration and inhibited the growth of transplanted tumors. Mechanistically, erianin may inhibit the progression of TNBC by downregulating the expression of SRC and interfering with cholesterol metabolism, indicating that erianin has the potential to be an effective treatment for TNBC. Moreover, this study provides a new perspective on the role of natural small molecules in treating malignant tumors.

**Table 1:** Primers for cholesterol synthesis and absorption-related genes

Primer name	Forward Primer(5’to3’)	Reverse Primer(5’to3’)	Species
HMGCR	TGATTGACCTTTCCAGAGCAAG	CTAAAATTGCCATTCCACGAGC	Human
HMGCR	TCTGTTGTGAACCATGTGACTTC	AGCTTGCCCGAATTGTATGTG	Mouse
LDLR	ACGGCGTCTCTTCCTATGACA	CCCTTGGTATCCCCAACAGA	Human
LDLR	TGACTCAGACGAACAAGGCTG	ATCTAGGCAATCTCGGTCTCC	Mouse
SREBP2	CCCTTCAGTGCAACGGTCATTCAC	TGCCATTGGCCGTTTGTGTC	Human
SREBP2	GCGTTCTGGAGACCATGGA	ACAAAGTTGCTCTGAAAACAAATCA	Mouse
DHCR24	GCACAGGCATCGAGTCATCAT	GTGCATCGCACAAAGCTGC	Human
DHCR24	GAGGCAGCTGGAGAAGTTTG	CCTTGCAGATCTTGTCGTAC	Mouse
SRC	GACAGGCTACATCCCCAGC	CGTCTGGTGATCTTGCCAAAA	Human
SRC	CAATGCCAAGGGCCTAAATGT	TGTTTGGAGTAGTAAGCCACGA	Mouse
GAPDH	GTCTTCACCACCATGGAGAAGGC	TTGTTGTCATGGATGACCTTGGCC	Mouse/Human

**Table 2:** KEGG Signaling Pathway Analysis (TOP15)

	KEGGID	Description	GeneRatio	BgRatio	pvalue	padj	geneName	Count
1	mmu00100	Steroid biosynthesis	6/368	23/15,107	1.43E-05	0.003934233	Dhcr24/Msmo1/Sqle/Cyp51/Fdft1/Cyp24a1	6
2	mmu00620	Pyruvate metabolism	10/368	102/15,107	0.000189582	0.026162271	Fh1/Ldhb/Acat2/Pcx/Acss2/Pdhb/Mdh1/Acaca/Ldha-ps2/Aldh2	10
3	mmu00561	Glycerolipid metabolism	9/368	89/15,107	0.000312855	0.02878263	Mogat2/Mboat2/Gpam/Lipg/Lpin1/Pnpla3/Aldh2/Dgkh/Plpp5	9
4	mmu00020	Citrate cycle (TCA cycle)	6/368	46/15,107	0.000824997	0.047030591	Fh1/Idh3b/Pcx/Idh3g/Pdhb/Mdh1	6
5	mmu01212	Fatty acid metabolism	8/368	82/15,107	0.000852003	0.047030591	Acat2/Gm13910/Scd1/Fasn/Cpt1a/Elovl6/Acaca/Hadhb	8
6	mmu00640	Propanoate metabolism	7/368	74/15,107	0.002131924	0.09806849	Ldhb/Acat2/Acss2/Acaca/Ldha-ps2/Bckdhb/Aldh6a1	7
7	mmu00230	Purine metabolism	13/368	223/15,107	0.003295604	0.129940974	Enpp4/Urah/Gucy1b2/Pnp2/Pde1b/Adcy1/Pnp/Nudt9/Nudt5/Gucy1a1/Ak5/Pde4b/Dck	13
8	mmu04625	C-type lectin receptor signaling pathway	10/368	151/15,107	0.003911808	0.134957378	Cd209a/Mras/Cd209e/Pycard/Il17d/Cd209d/Cd209b/Nfatc4/Rras/Cd209g	10
9	mmu00280	Valine, leucine and isoleucine degradation	6/368	66/15,107	0.005330241	0.163460719	Acat2/Gm13910/Hadhb/Bckdhb/Aldh2/Aldh6a1	6
10	mmu05202	Transcriptional misregulation in cancer	16/368	363/15,107	0.016606876	0.458349778	Nupr1/Hoxa9/Met/Mlf1/Ldb1/Gadd45a/Gm12183/Hpgd/Bcl2l1/Mmp3/Prom1/Gm37486/H3f3a-ps1/Igfbp3/Gm26626/Bcl2a1b	16
11	mmu04974	Protein digestion and absorption	7/368	112/15,107	0.019685905	0.490787516	Slc6a19/Col27a1/Cpa3/Kcnq1/Col6a6/Col7a1/Col2a1	7
12	mmu04928	Parathyroid hormone synthesis, secretion and action	8/368	140/15,107	0.021338588	0.490787516	Vdr/Adcy1/Creb3l4/Slc9a3r1/Casr/Pde4b/Cyp24a1/Mmp14	8
13	mmu00071	Fatty acid degradation	5/368	67/15,107	0.023528136	0.491004434	Acat2/Gm13910/Cpt1a/Hadhb/Aldh2	5
14	mmu03460	Fanconi anemia pathway	5/368	68/15,107	0.024906022	0.491004434	Fancd2/Hes1/Fancl/Brip1/Brca1	5
15	mmu00564	Glycerophospholipid metabolism	7/368	132/15,107	0.042722755	0.74014812	Mboat2/Gpam/Pla1a/Lpin1/Pla2g2d/Dgkh/Plpp5	7

## Data Availability

No datasets were generated or analysed during the current study.

## Abbreviations

TNBC: triple-negative breast cancer
Her-2: human epidermal growth factor receptor 2
BC: breast cancer
CETSA: cellular thermal shift assay
DEGs: differentially expressed genes
WB: Western blotting
IHC: immunohistochemistry
FBS: fetal bovine serum
PBS: phosphate buffer saline
OD: optical density
BSA: bovine serum albumin
HE: hematoxylin and eosin
DMSO: dimethyl sulfoxide
TCGA: The Cancer Genome Atlas
GOT: glutamic oxaloacetic transaminase
GPT: glutamic pyruvic transaminase
